# Knowledge Mapping of Necroptosis From 2012 to 2021: A Bibliometric Analysis

**DOI:** 10.3389/fimmu.2022.917155

**Published:** 2022-06-13

**Authors:** Jie Zhang, Luxia Song, Jundi Jia, Wende Tian, Runmin Lai, Zihao Zhang, Jingen Li, Jianqing Ju, Hao Xu

**Affiliations:** ^1^ National Clinical Research Center for Chinese Medicine Cardiology, Xiyuan Hospital, China Academy of Chinese Medical Sciences, Beijing, China; ^2^ Graduate School, Beijing University of Chinese Medicine, Beijing, China; ^3^ Graduate School, China Academy of Chinese Medical Sciences, Beijing, China; ^4^ Department of Cardiovascular Medicine, Dongzhimen Hospital, Beijing University of Chinese Medicine, Beijing, China

**Keywords:** necroptosis, programmed cell death, CiteSpace, VOSviewer, bibliometric

## Abstract

**Background:**

Necroptosis, a recently discovered programmed cell death, has been pathologically linked to various diseases and is thus a promising target for treating diseases. However, a comprehensive and objective report on the current status of entire necroptosis research is lacking. Therefore, this study aims to conduct a bibliometric analysis to quantify and identify the status quo and trending issues of necroptosis research in the last decade.

**Methods:**

Articles were acquired from the Web of Science Core Collection database. We used two bibliometric tools (CiteSpace and VOSviewer) to quantify and identify the individual impact and cooperation information by analyzing annual publications, journals, co-cited journals, countries/regions, institutions, authors, and co-cited authors. Afterwards, we identified the trending research areas of necroptosis by analyzing the co-occurrence and burst of keywords and co-cited references.

**Results:**

From 2012 to 2021, a total of 3,111 research articles on necroptosis were published in 786 academic journals by 19,687 authors in 885 institutions from 82 countries/regions. The majority of publications were from China and the United States, of which the United States maintained the dominant position in necroptosis research; meanwhile, the Chinese Academy of Sciences and Ghent University were the most active institutions. Peter Vandenabeele published the most papers, while Alexei Degterev had the most co-citations. *Cell Death & Disease* published the most papers on necroptosis, while *Cell* was the top 1 co-cited journal, and the major area of these publications was molecular, biology, and immunology. High-frequency keywords mainly included those that are molecularly related (MLKL, TNF-alpha, NF-κB, RIPK3, RIPK1), pathological process related (cell-death, apoptosis, necroptosis, necrosis, inflammation), and disease related (cancer, ischemia/reperfusion injury, infection, carcinoma, Alzheimer’s disease).

**Conclusion:**

Necroptosis research had a stable stepwise growth in the past decade. Current necroptosis studies focused on its cross-talk with other types of cell death, potential applications in disease treatment, and further mechanisms. Among them, the synergy with ferroptosis, further RIPK1/RIPK3/MLKL studies, its association with inflammation and oxidative stress and translational applications, and the therapeutic potential to treat cancer and neurodegenerative diseases are the trending research area. These might provide ideas for further research in the necroptosis field.

## 1 Introduction

Necroptosis is a form of programmed cell death (PCD) mediated by receptor interacting protein kinase-3 (RIPK3) and its substrate mixed lineage kinase domain-like protein (MLKL) (1). It was first observed in 1988 as a tumor necrosis factor (TNF)–induced new necrotic cell death, with the “classical” features of apoptosis but a “balloon-like” morphology without unclear disintegration (2). In 2005, Junying Yuan and colleagues proposed the term “necroptosis” and identified a specific small-molecule inhibitor of necroptosis, necrostatin-1, which blocks a critical step in necroptosis (3). Necroptosis is morphologically similar to necrosis but rigorously regulated by intracellular signaling cascades. Apoptosis and necroptosis are both PCD; compared to the former, necroptosis is an inflammatory process characterized by swollen organelles, membrane pores, and the eventual rupture of the plasma membrane and organelles (4). The initiation of necroptosis is usually triggered by the activation of death receptors (e.g., TNF receptor), which subsequently recruit receptor interacting protein kinase-1 (RIPK1). The elimination or inhibition of caspase-8 enables the activation of RIPK1 and recruitment of RIPK3. RIPK3 then recruits and phosphorylates MLKL, and phosphorylated MLKL oligomerizes and moves to the plasma membrane and forms a pore, leading to the membrane rupture (1, 2). Necroptosis has been pathologically related to various human diseases, such as cancers (5–7), Alzheimer’s disease (AD) (8), Parkinson’s disease (PD) (9), multiple sclerosis (10), stroke (11), infection (12), inflammatory bowel disease (13), pancreatitis (14), and atherosclerosis (15). Consequently, necroptosis is considered to be a promising target for many diseases.

According to its great potential, necroptosis has gained researchers’ keen interest with a rapidly increasing number of publications. Many reviews have summarized necroptosis studies from various aspects (7, 16–20), including a bibliometric analysis (21); however, this bibliometric study only focused on neuroscience and included both research articles and reviews. Therefore, to the best of our knowledge, there is no report on the whole picture of necroptosis research.

Although a quantitative overview could be conducted through many approaches, such as traditional review, systematic review, main path analysis, evidence map, and bibliometrics (22), only bibliometrics could qualitatively and quantitatively analyze the contribution and cooperation of authors, institutions, countries, and journals and evaluate the knowledge base and trending research topics at the same time (23, 24).

Therefore, the present study aims to use two bibliometric software, CiteSpace and VOSviewer, to quantify the whole picture of necroptosis research and identify trending research questions in the last decade, which may help to generate hypotheses for future studies in the necroptosis field.

## 2 Materials and Methods

### 2.1 Data Collection

The Web of Science Core Collection (WoSCC) database is widely used in bibliometrics, which contains Science Citation Index Expanded (SCIE), Social Science Citation Index (SSCI), and Emerging Sources Citation Index (ESCI) (21, 24). Data were obtained from the WoSCC database on March 24, 2022. The search formula was [TS = (“necroptosis” OR “necroptotic”)] AND [Publication type = (Article)] AND [Language = (English)], and the publication year was limited to (2012-2021). Search results were downloaded as “Full Record and Cited References” and “Plain Text”. For further analysis, we subsequently renamed the files as “download_*.txt”, which CiteSpace software could read.

### 2.2 Data Analysis

We used CiteSpace 5.8.R3 (Chaomei Chen, 2006), VOSviewer 1.6.16 (Nees Jan van Eck and Ludo Waltman, 2010), and Microsoft Excel 2019 to perform bibliometric analysis and visualization. Data cleaning was the first step, for instance, “tumor cell” and “tumor-cells” were merged as “tumor cells”, “reactive oxygen” and “ros” were unified as “ros”, and meaningless terms such as “age” and “assay” were deleted (25).

CiteSpace is a bibliometric and visual analysis tool that excels at detecting cooperation, key points, internal structure, potential trends, and dynamics in a scientist field (26). Therefore, we used CiteSpace to analyze the co-occurrence of countries/regions and institutions, the dual-map of journals, reference timeline, citation bursts, keyword timeline, and keyword bursts. The settings were as follows: timespan (2012–2021), years per slice (1), pruning (none), selection criteria (Top N=100), minimum duration of burstness (2 years), cluster labels were extracted by light semantic indexing (LSI) and the log-likelihood ratio (LLR) algorithm, and others followed the default. In CiteSpace visualization, the size of node reflects the co-occurrence frequencies, and the links indicate the co-occurrence relationships. The colors of the node and line represent different years; the colors vary from purple to red as time goes from 2012 to 2021. Nodes with purple round mean a high betweenness centrality (≥0.10), which acts as a bridge between different networks (26–28).

VOSviewer is another bibliometric software that is good at creating and visualizing knowledge maps, showing the types of clusters, overlays, or density colors (29, 30). It was used to perform the co-occurrence of authors and co-cited authors, journals and co-cited journals, co-cited references, and keywords. We set the counting method as full counting; other thresholds were shown in the corresponding chapter. In the cluster map, the size of node reflects the co-occurrence frequencies, and the same color represents the same cluster; furthermore, the link indicates the co-occurrence relationship, and the thickness of the link depends on a calculated strength value, which is proportional to the number of publications two researchers co-authored or the number of publications in which two keywords occur together (30). In density maps, the size of word and round and the opacity of yellow are positively related to the co-cited frequency. In the overlay map, the color indicates the average published year.

We used Excel software to analyze the annual publications. Furthermore, the impact factor (IF) and Journal citation reports (JCR) division of journals and the H-index of scholars were obtained from the Web of Science on April 5, 2022.

## 3 Results

### 3.1 Annual Growth Trend

**Figure 1:**
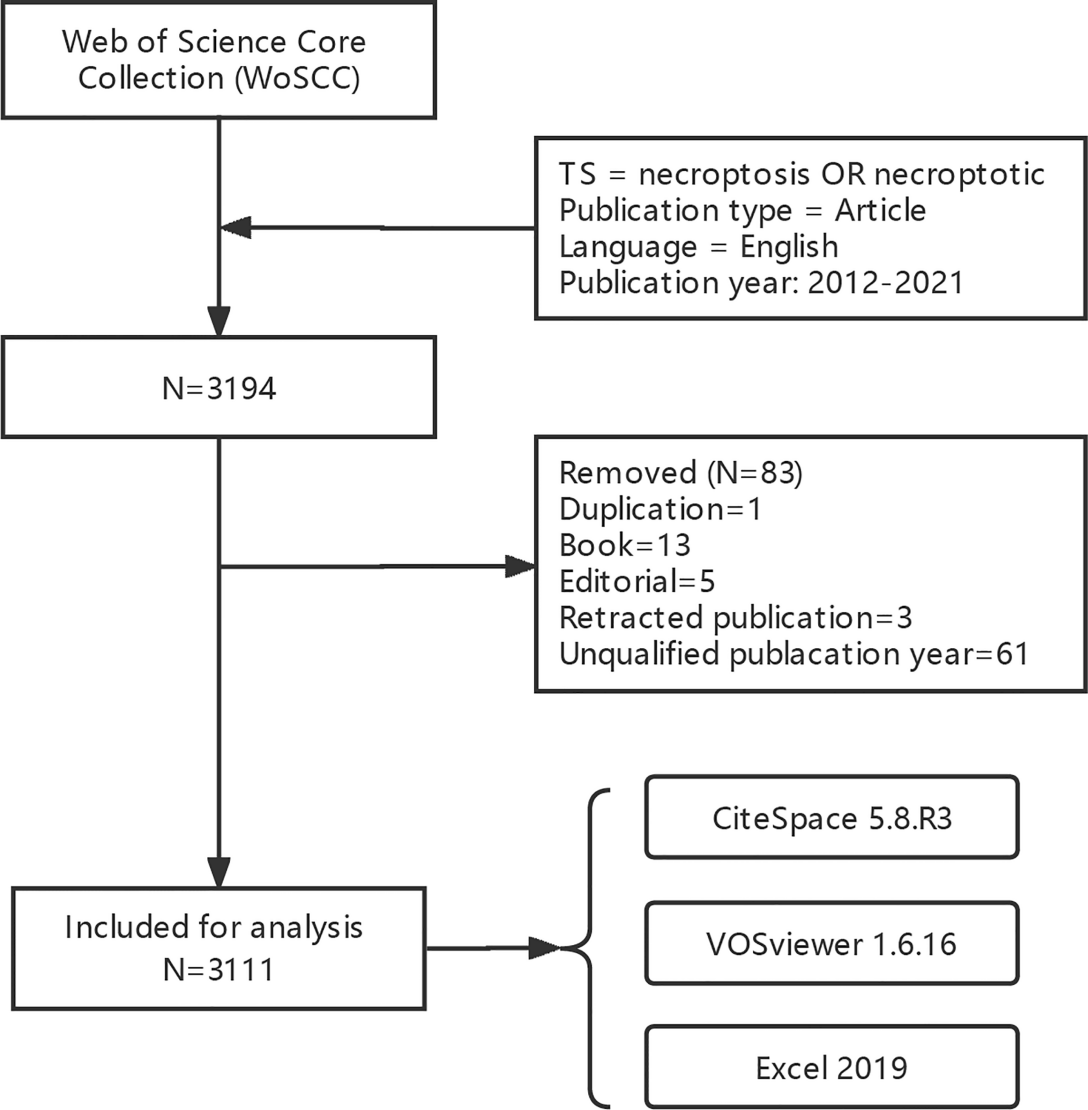
Flow chart of data collection.

**Figure 2:**
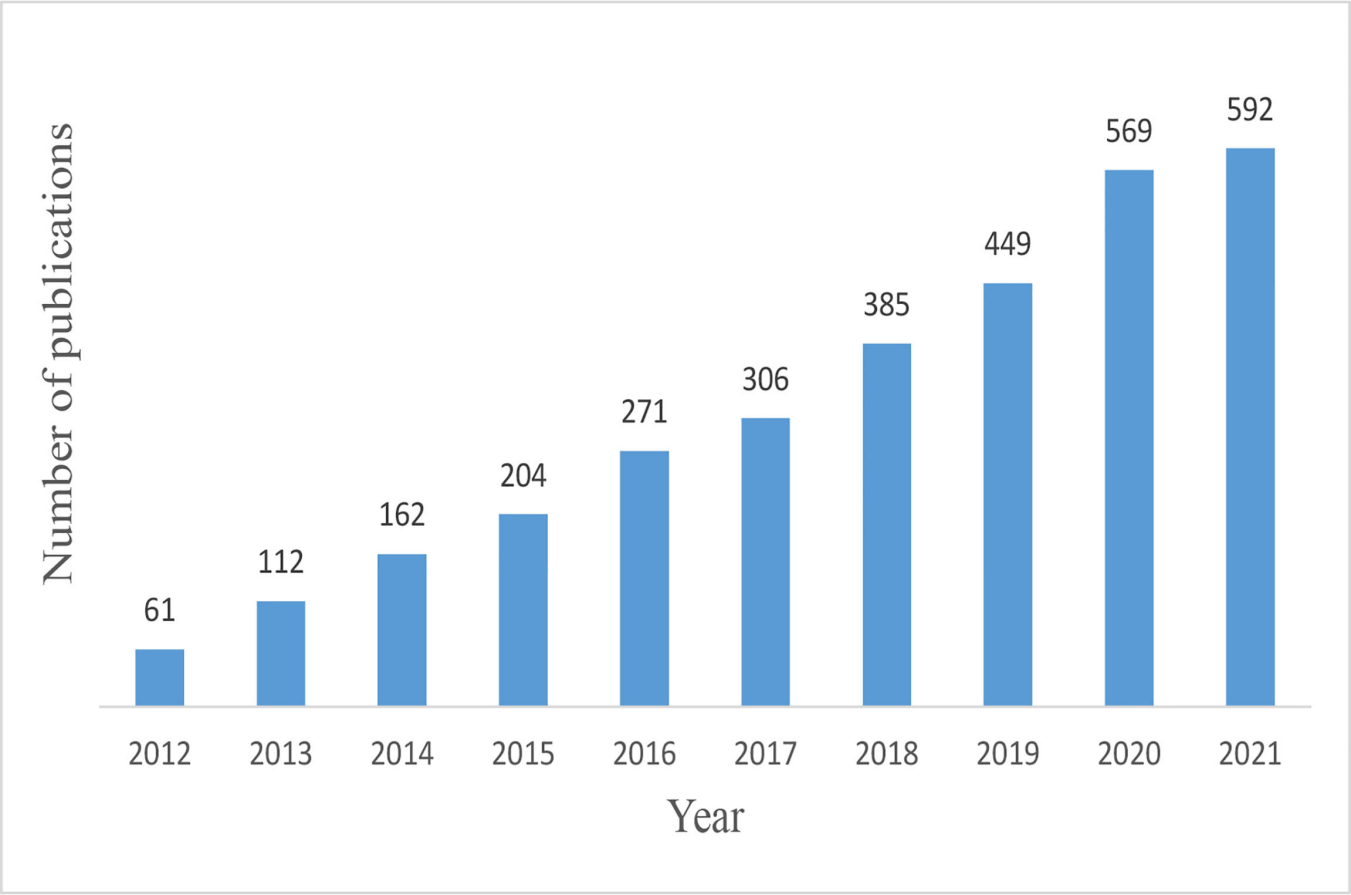
Trends of necroptosis publications over the past decade.

We obtained 3,194 papers from the WoSCC database and finally included 3,111 eligible articles (**Figure 1**; **Supplementary Table 1**). As shown in **Figure 2**, the number of necroptosis-related articles has steadily increased over the past decade.

### 3.2 Distribution of Countries/Regions and Institutions

**Figure 3:**
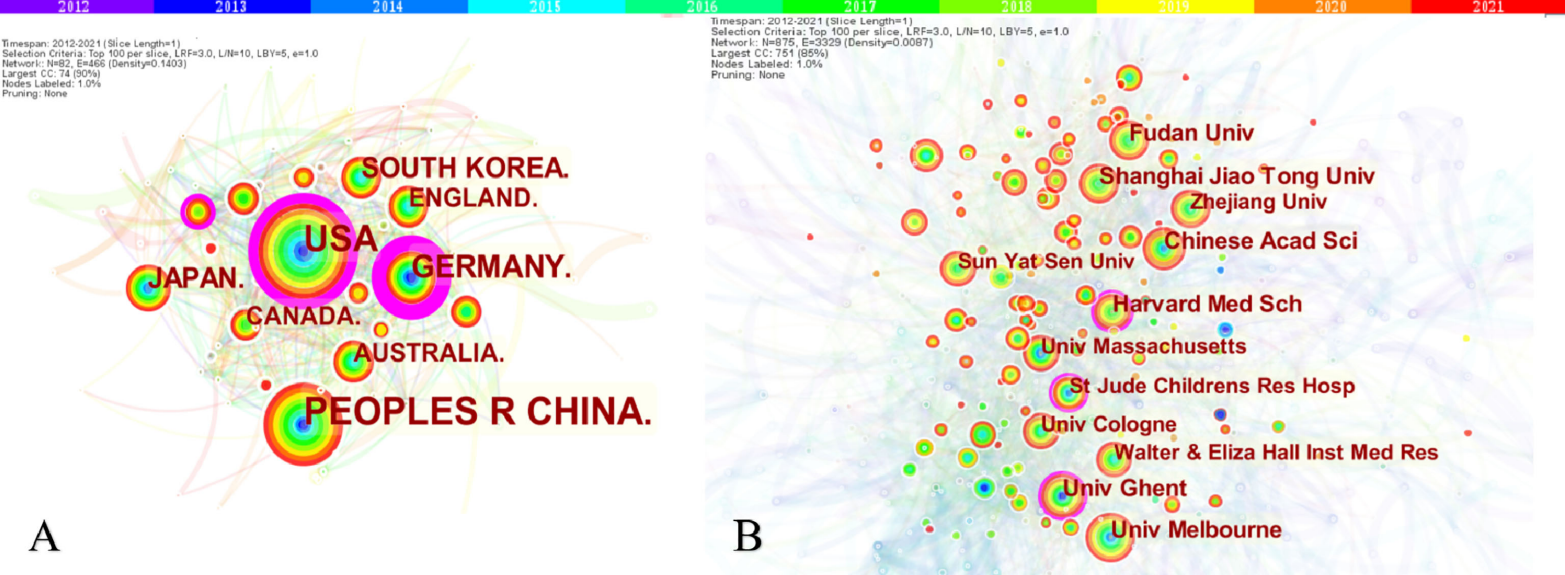
The co-occurrence map of countries/regions **(A)** and institutions **(B)** in necroptosis research. **(A)** Country/regions (n≥100); **(B)** Institution (n≥50). The node size reflects the co-occurrence frequencies, and the links indicate the co-occurrence relationships. The color of node and line represent different years; colors vary from purple to red as time goes from 2012 to 2021, and nodes with purple round mean high betweenness centrality (≥0.1).

A total of 3,111 papers were from 82 different countries/regions and 885 institutions (**Table 1**). China published the most articles (n=1,213), followed by the United States (n=955) and Germany (n=318). However, the centrality of China was less than 0.10, which means China might not be a “bridge” node in necroptosis studies. By contrast, Germany (centrality=0.41), the United States (centrality=0.23), and France (centrality=0.14) had high centrality, which is presented with a purple circle in **Figure 3A**. The density of country/region co-occurrence (**Figure 3B**) was 0.14, indicating active cooperation among them. Chinese Academy of Sciences is the most productive institution, but its centrality is relatively low (n=86, centrality=0.07). By contrast, Harvard Medical School (n=70, centrality=0.14), Ghent University (n=77, centrality=0.12), and St. Jude Children’s Research Hospital (n=55, centrality=0.10) had a high centrality.

**Table 1:** Top 10 countries/regions and institutions related to necroptosis.

Rank	Countries/Regions	Centrality	Count	Institution	Centrality	Count
1	China	0.06	1213	Chinese Acad Sci (China)	0.07	86
2	United States	0.23	955	Univ Ghent (Belgium)	0.12	77
3	Germany	0.41	318	Univ Melbourne (Australia)	0.07	74
4	Japan	0.07	195	Harvard Med Sch (United States)	0.14	70
5	South Korea	0.09	172	Fudan Univ (China)	0.07	67
6	England	0.09	130	Shanghai Jiao Tong Univ (China)	0.06	65
7	Australia	0.08	116	Zhejiang Univ (China)	0.08	62
8	Canada	0.05	116	Walter & Eliza Hall Inst Med Res (Australia)	0.04	60
9	Belgium	0.08	97	Sun Yat Sen Univ (China)	0.06	58
10	France	0.14	94	St Jude Childrens Res Hosp (USA)	0.10	55

### 3.3 Authors and Co-Cited Authors

**Figure 4:**
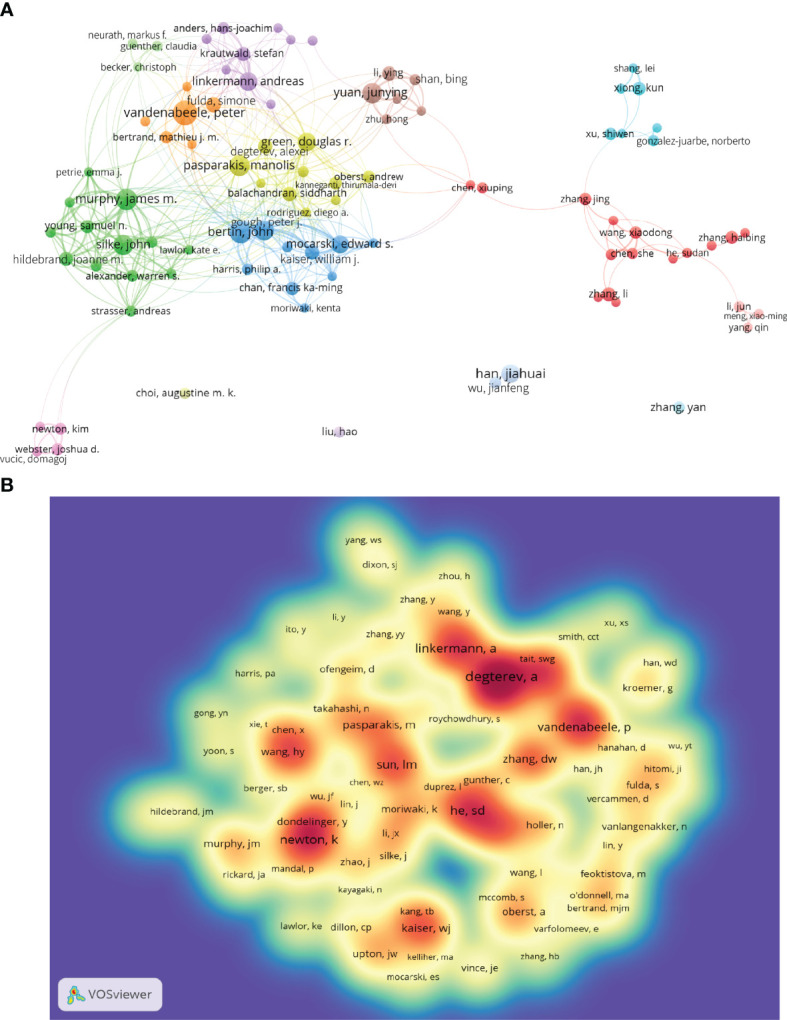
The co-occurrence authors’ **(A)** and co-cited authors’ **(B)** map of necroptosis research. **(A)** Authors with documents ≥10 (cluster map). The size of node reflects the authors’ co-occurrence frequencies, the link indicates the co-occurrence relationship between authors, the thickness of the link is proportional to the number of publications two researchers have co-authored, and the same color of node represents the same cluster. **(B)** Co-cited authors with citations ≥100 (density map). The size of word, the size of round, and the opacity of yellow are positively related to the co-cited frequency.

A total of 19,687 authors were involved in necroptosis research; among them, 91 authors published at least ten papers (**Figure 4A**; **Supplementary Table 2**). Peter Vandenabeele from the University of Ghent published the highest number of necroptosis-related articles (n=45), followed by John Bertin and James M. Murphy (**Table 2**). There were fifteen colors in **Figure 4A**, representing 15 clusters among authors. Active collaborations usually exist in the same cluster, such as Bertin John and Gough Peter J. There were also collaborations among linked two nodes in different clusters, such as John Bertin and Peter Vandenabeele.

**Table 2:** Top 10 authors and co-cited authors related to necroptosis.

Rank	Author	Count	H-index	Co-cited author	Count	H-index
1	Peter Vandenabeele	45	118	Alexei Degterev	1363	40
2	John Bertin	37	66	Sudan He	866	20
3	James M. Murphy	36	18	Kim Newton	865	39
4	Manolis Pasparakis	34	76	Andreas Linkermann	843	47
5	Junying Yuan	33	63	Galluzzi L	765	107
6	Peter J. Gough	32	38	Liming Sun	752	14
7	John Silke	32	72	Peter Vandenabeele	742	118
8	Douglas R. Green	29	23	Young-Sik Cho	705	19
9	Andreas Linkermann	29	47	William J. Kaiser	616	51
10	Edward S. Mocarski	27	82	Duanwu Zhang	597	11

Co-cited authors are the authors who were cited in one article (31). Among 61,882 co-cited authors, 99 had over 100 co-citations (**Figure 4B**; **Supplementary Table 2**). **Figure 4B** presented them as a density map, which could clearly show the high-frequency co-cited authors. The more citations, the warmer the color. As shown in **Table 2** and **Figure 4B**, Alexei Degterev, Sudan He, and Kim Newton had the most co-cited.

Given the inherent limitations of the CiteSpace and VOSviewer visualization, the pictures cannot show all the information. Therefore, we placed the complete data in **Supplementary Table 2**, as well as the figures below.

### 3.4 Journals and Co-Cited Academic Journals

A total of 786 academic journals published articles on necroptosis research. The top 15 journals published 821 papers, accounting for 26.39% of all publications (**Table 3**). *Cell Death & Disease* had the greatest number of publications (n=164), followed by *Cell Death and Differentiation* (n=87) and *Scientific Reports* (n=84).

**Table 3:** Top 15 journals and co-cited journals related to necroptosis.

Rank	Journal	Count	JCR(2020)	IF(2020)	Cited journal	Cited count	JCR(2020)	IF(2020)
1	Cell Death & Disease	164	Q1	8.469	Cell	6414	Q1	41.584
2	Cell death and Differentiation	87	Q1	15.828	Nature	5083	Q1	49.962
3	Scientific Reports	84	Q1	4.380	Proceedings of the national academy of sciences of the United States of America	4210	Q1	11.205
4	Proceedings of the National Academy of Sciences of the United States of America	55	Q1	11.205	Cell Death and Differentiation	4183	Q1	15.828
5	Plos One	51	Q2	3.240	Journal of Biological Chemistry	3941	Q2	5.157
6	Nature Communications	47	Q1	14.919	Science	2791	Q1	47.728
7	Cell Reports	46	Q1	9.423	Immunity	2472	Q1	31.745
8	International Journal of Molecular Sciences	45	Q1/Q2	5.924	Cell Death & Disease	2410	Q1	8.469
9	Biochemical and biophysical research communications	40	Q2/Q3	3.575	Molecular Cell	2299	Q1	17.970
10	Journal of Immunology	40	Q2	5.422	Plos One	2205	Q2	3.240
11	Oncotarget	40	/	/	Journal of Immunology	2148	Q2	5.422
12	Cell death Discovery	33	Q2	5.241	Nature Reviews Molecular Cell Biology	1812	Q1	94.444
13	Journal of Biological Chemistry	33	Q2	5.157	Nature Communications	1496	Q1	14.919
14	Frontiers in Immunology	30	Q1	7.561	Nature Chemical Biology	1396	Q1	15.040
15	Molecular Medicine Reports	26	Q3/Q4	2.952	Cell Reports	1335	Q1	9.423

Among 6,555 co-cited sources, 49 journals had >500 citations; among which, *Cell* (n=6,414), *Nature* (n=5,083), and *Proceedings of the National Academy of Sciences of the United States of America* (*PNAS*) (n=4,210) had the greatest number of citations. Furthermore, the top 15 co-cited journals accounted for 28.20% citation of all cited sources (**Table 3**).

**Figure 5:**
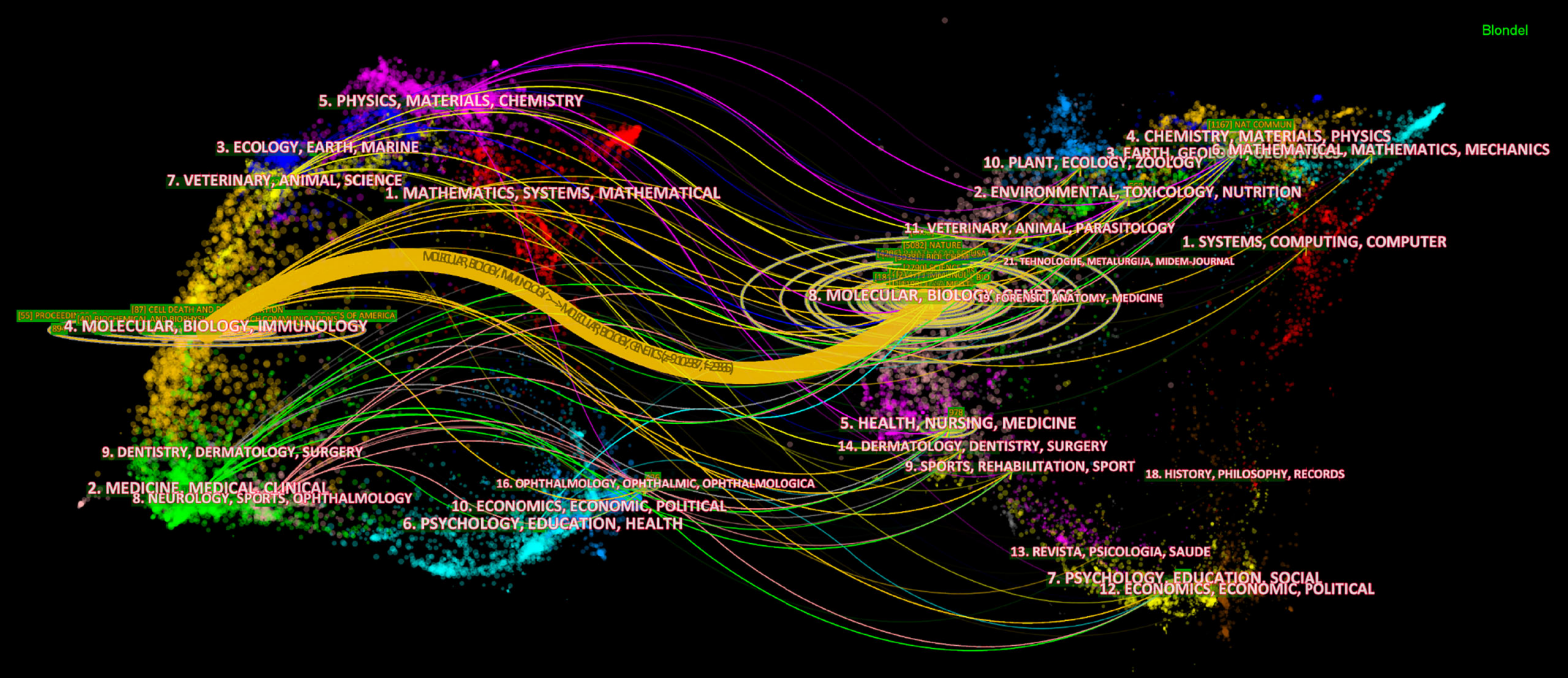
The dual-map overlay of journals on necroptosis. The citing journals are on the left, the cited journals are on the right, and the colored path represents the citation relationship.

The dual-map overlay of journals stands for the topic distribution of academic journals (32) (**Figure 5**). Citing journals are on the left, cited journals are on the right, and colored paths indicate citation relationships. **Figure 5** showed there was only one primary citation path from Molecular/Biology/Genetics journals to Molecular/Biology/Immunology journals.

### 3.5 Co-Cited References and Reference Burst

Of the 89,431 cited references, 71 were cited at least 100 times (**Supplementary Table 3**). **Table 4** showed that the top 10 co-cited references were co-cited at least 348 times. Among them, the most co-cited reference is an article published in *Cell* by Liming Sun et al. in 2011 (n=714). Furthermore, seven of the top 10 were research articles, two were reviews, and one was a report.

**Table 4:** Top 10 co-cited references related to necroptosis.

	Title	First Author	Journals	Citations	Type	Year
1	Mixed lineage kinase domain-like protein mediates necrosis signaling downstream of RIP3 kinase (33)	Liming Sun	Cell	714	Article	2011
2	Phosphorylation-driven assembly of the RIP1–RIP3 complex regulates programmed necrosis and virus-induced inflammation (34)	Young Sik Cho	Cell	657	Article	2009
3	Chemical inhibitor of nonapoptotic cell death with therapeutic potential for ischemic brain injury (3)	Alexei Degterev	Nat Chem Biol	652	Article	2005
4	Receptor Interacting Protein Kinase-3 Determines Cellular Necrotic Response to TNF-a (35)	Sudan He	Cell	647	Article	2009
5	Molecular mechanisms of necroptosis: an ordered cellular explosion (36)	Peter Vandenabeele	Nat Rev Mol Cell Biol	542	Review	2010
6	RIP3, an energy metabolism regulator that switches TNF-induced cell death from apoptosis to necrosis (37)	Duan-Wu Zhang	Science	539	Report	2009
7	Identification of RIP1 kinase as a specific cellular target of necrostatins (38)	Alexei Degterev	Nat Chem Biol	520	Article	2008
8	Necroptosis and its role in inflammation (17)	Manolis Pasparakis	Nature	463	Review	2015
9	Mixed lineage kinase domain–like protein MLKL causes necrotic membrane disruption upon phosphorylation by RIP3 (39)	Huayi Wang	Mol Cell	438	Article	2014
10	Plasma membrane translocation of trimerized MLKL protein is required for TNF-induced necroptosis (40)	Zhenyu Cai	Nat Cell Biol	348	Article	2014

**Figure 6:**
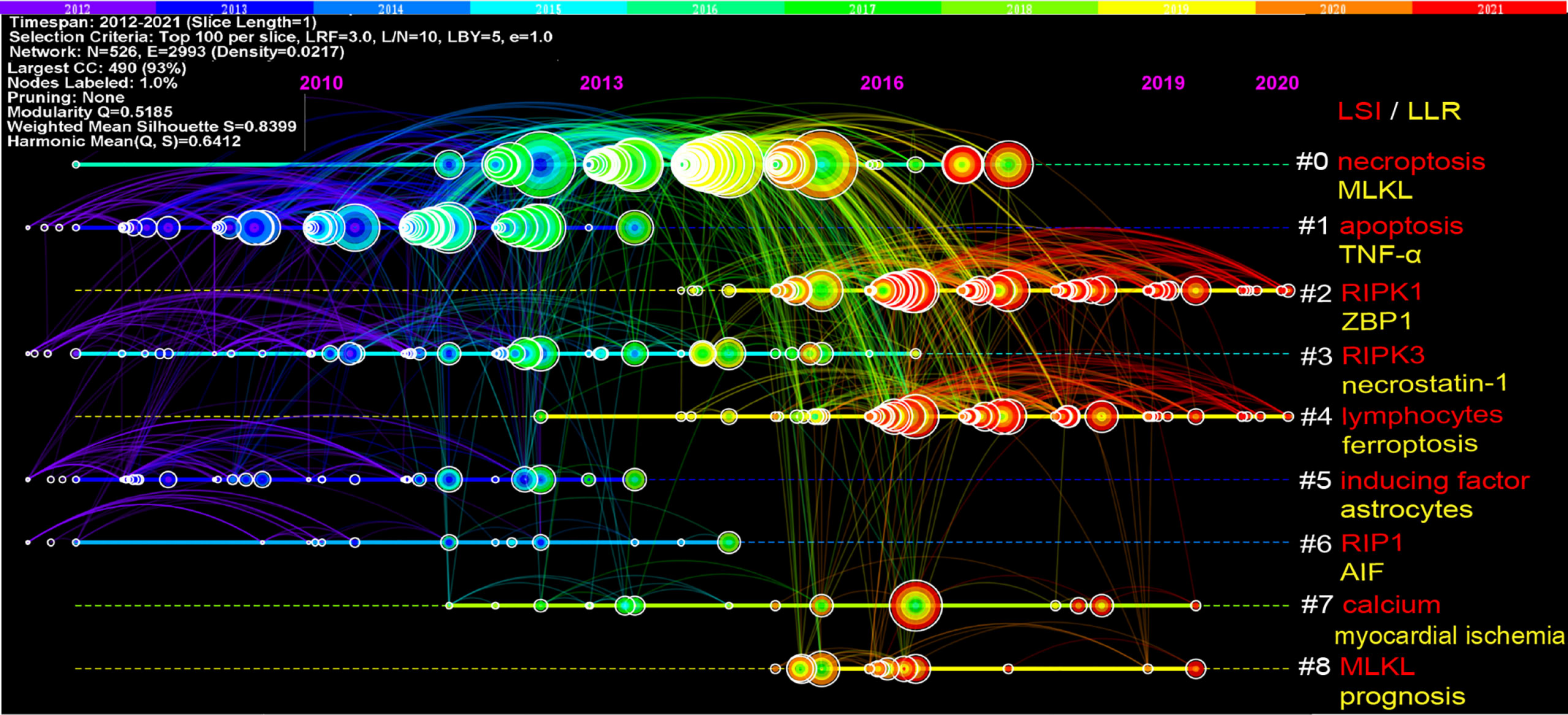
Timeline view of co-cited references related to necroptosis. Each horizontal line represents a cluster; the smaller the number, the larger the cluster, and #0 is the largest cluster. The node size reflects the co-cited frequencies, and the links indicate the co-cited relationships; the color of node and line represent different years; nodes are at their first co-cited year. Cluster labels were extracted from title by LSI (red) and LLR (yellow).

The references timeline view could visualize the evolution of research hotspots over time. The terms with the highest frequency in each cluster were tagged as cluster labels, and the rest were listed in **Supplementary Table 2**. As shown in **Figure 6**, cluster #0 (necroptosis/MLKL), #1 (apoptosis/TNF-α), #3 (RIPK3/necrostatin-1), #5 (inducing factor/astrocytes), and #6 (RIP1/AIF) started earlier; while cluster #2 (RIPK1/ZBP1) and #4 (lymphocytes/ferroptosis) are still ongoing, which could be considered as the frontier.

**Figure 7:**
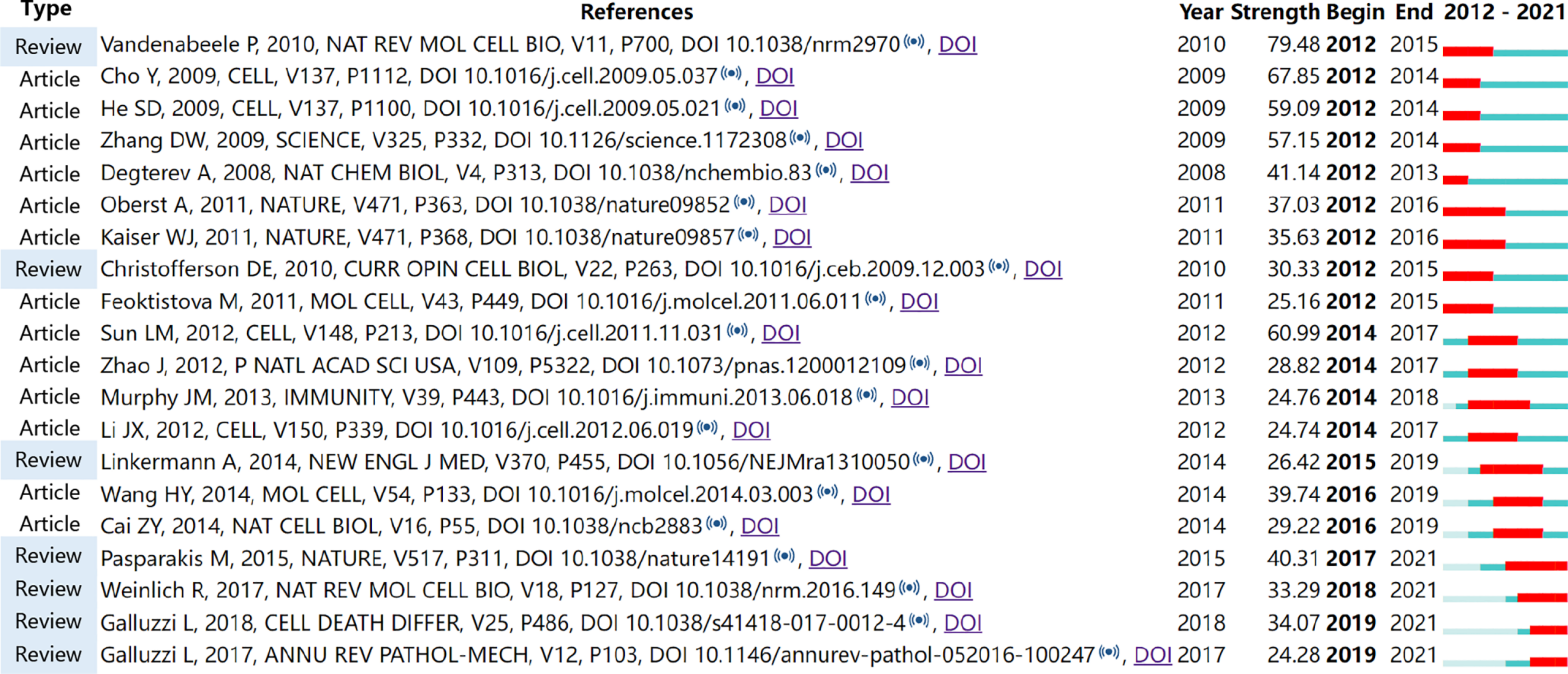
Top 20 references with the strongest citation bursts involved in necroptosis (sorted by the starting year). The Blue bars mean the reference had been published; the red bars represent citation burstness.

References with citation bursts are those that have been cited significantly more frequently over a period (28). A total of 243 references were detected as citation bursts, and we listed the top 20 in **Figure 7**. The strongest burstness (strength=79.48) occurred in a paper entitled “Molecular mechanisms of necroptosis: an ordered cellular explosion” (36), published in *Nat Rev Mol Cell Biol* by Vandenabeele et al. in 2010, with citation burstness from 2012 to 2015. Notably, four references (16–18, 41) were still in burstness. Respectively, Pasparakis et al. (17) reviewed necroptosis and its role in inflammation; Weinlich et al. reviewed necroptosis in development, inflammation, and disease (18); the Nomenclature Committee on Cell Death prompted a recommendation on the molecular mechanisms of cell death (41); Galluzzi et al. (16) reviewed the mechanisms of necroptosis and its relevance to disease.

### 3.6 Keyword Analysis of Trending Research Topic

A total of 5,639 keywords were extracted, among which 297 keywords appeared at least ten times and 62 keywords appeared at least 50 times. As we can see from **Table 5**, cell death was the most frequent keyword (n=1,211), followed by apoptosis (n=1,104) and necroptosis (n=885). **Table 6** showed the top 10 keywords of molecules, pathological processes, and diseases related to necroptosis. It could be seen that MLKL (n=521), TNF-alpha (n=332), NF-κB (n=315), RIPK3 (n=233), and RIPK1 (n=155) were the most studied molecules; cell-death (n=1211), apoptosis (n=1,104), necroptosis (n=885), necrosis (n=557), inflammation (n=443), and oxidative stress (n=273) were the most mentioned pathological process; and cancer (n=170), ischemia/reperfusion injury (n=110), infection (n=50), carcinoma (n=32), and Alzheimer’s disease (n=31) were the most studied diseases in necroptosis studies.

**Table 5:** Top 20 keywords related to necroptosis.

Rank	Keywords	Counts	Rank	Keywords	Counts
1	cell-death	1,211	11	TNF-alpha	358
2	apoptosis	1,104	12	NF-κB	315
3	necroptosis	885	13	oxidative stress	273
4	activation	711	14	kinase	259
5	necrosis	557	15	phosphorylation	254
6	MLKL	521	16	inhibition	238
7	programmed necrosis	467	17	RIPK3	233
8	inflammation	443	18	autophagy	204
9	mechanism	393	19	pathway	194
10	expression	391	20	cancer	170

MLKL, mixed lineage kinase domain-like protein; TNF-alpha, tumor necrosis factor-alpha; NF-κB, nuclear factor κ-light-chain-enhancer of activated B cells; RIPK3, receptor-interacting protein kinase-3.

**Table 6:** Top 15 molecules, pathological process and disease related to necroptosis.

Rank	Molecules	Counts	Pathological process	Counts	Diseases	Counts
1	MLKL	521	Cell-death	1,211	Cancer	170
2	TNF-alpha	358	Apoptosis	1,104	Ischemia/reperfusion injury	110
3	NF-κB	315	Necroptosis	885	Infection	50
4	RIPK3	233	Necrosis	557	Carcinoma	32
5	RIPK1	155	Inflammation	443	Alzheimer’s disease	31
6	Complex	89	Oxidative stress	273	Brain injury	30
7	Caspase-8	82	Phosphorylation	254	Fibrosis	26
8	NLRP3	65	Autophagy	204	Stroke	26
9	ROS	50	Proliferation	70	Acute kidney injury	25
10	FADD	38	ER stress	65	Breast cancer	25
11	Necrostatin-1	37	Ubiquitination	45	Tumor	23
12	Inflammasome	34	DNA damage	40	Drug resistance	22
13	p53	33	Pyroptosis	26	Hepatocellular carcinoma	22
14	bcl-2	32	Ferroptosis	25	Inflammatory bowel disease	20
15	Toll-like receptors	19	Mitophagy	19	Parkinson’s disease	19

MLKL, mixed lineage kinase domain-like protein; TNF-alpha, tumor necrosis factor-alpha; NF-κB, nuclear factor κ-light-chain-enhancer of activated B cells; RIPK3, receptor-interacting protein kinase-3; RIPK1, receptor-interacting protein kinase-1; NLRP3, NOD-, LRR- and pyrin domain-containing protein 3; ROS, reactive oxygen species; FADD, Fas-associated via death domain; er stress, endoplasmic reticulum stress; bcl-2, B-cell lymphoma-2.

**Figure 8:**
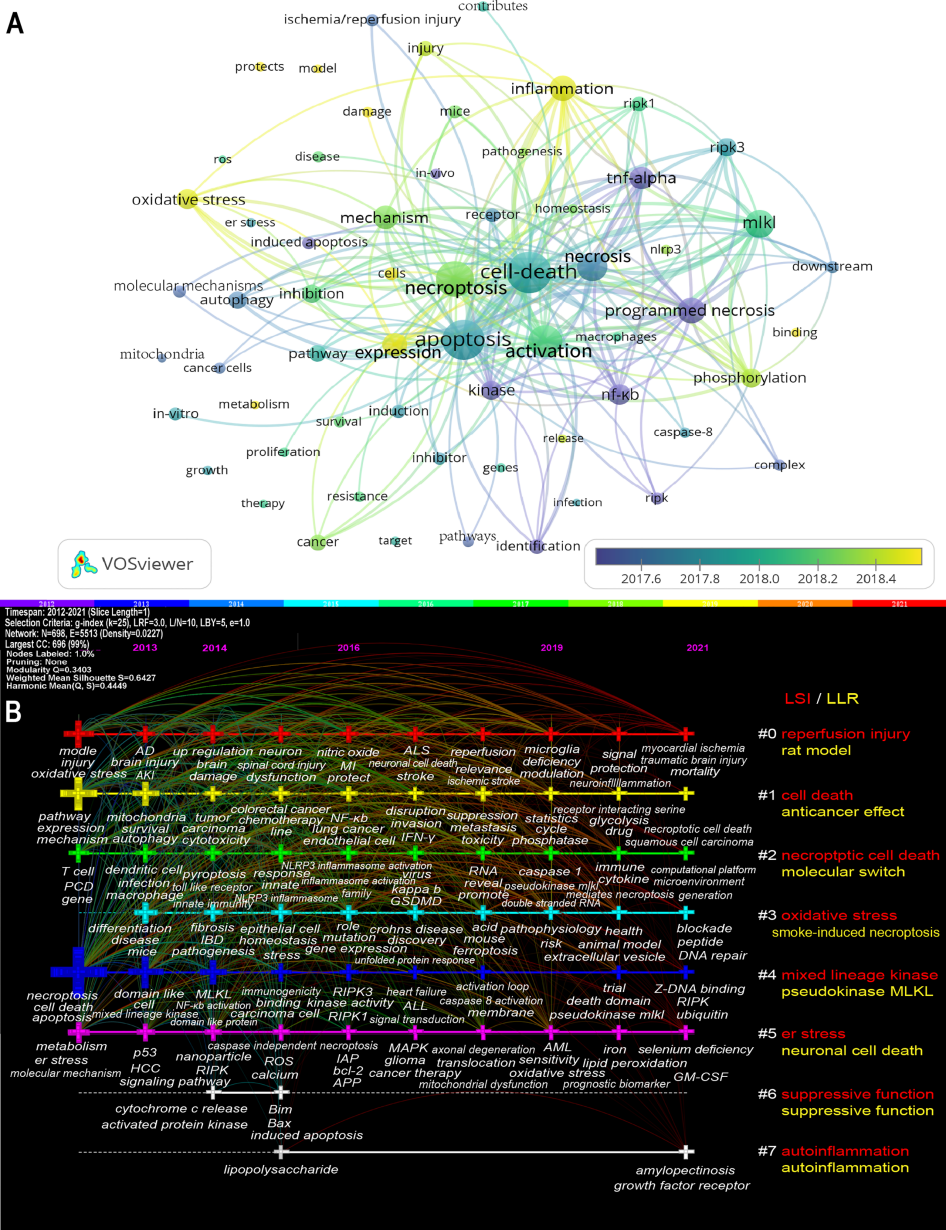
The overlay map **(A)** and timeline view **(B)** of keywords related to necroptosis. **(A)** Keywords appeared ≥50, max lines = 200. The node size reflects the co-occurrence frequencies, the link indicates the co-occurrence relationship, the thickness of the link is proportional to the number of times two keywords co-occur, and the color indicates the average published year. **(B)** Each horizontal line represents a cluster; the smaller the number, the larger the cluster, and #0 is the largest. The time is at the top, and keywords are located at their first co-occurrence time in the cluster. Cluster labels were extracted from title and abstract information by LSI (red) and LLR (yellow). LSI, light semantic indexing; LLR, log-likelihood ratio; AD, Alzheimer’s disease; AKI, acute kidney injury; ALL, acute lymphoblastic leukemia; ALS, amyotrophic lateral sclerosis; AML, Acute Myelocytic leukemia; APP, amyloid precursor protein; bcl-2, B-cell lymphoma-2; er stress, endoplasmic reticulum stress; GM-CSF, granulocyte/macrophage colony-stimulating factor; GSDMD, gasdermin-D; HCC, hepatocellular carcinoma; IAP, inhibitors of apoptosis; IBD, inflammatory bowel disease; INF-γ, interferon-γ; MI, myocardial infarction; MLKL, mixed lineage kinase domain-like protein; NF-κB, nuclear factor κ-light-chain-enhancer of activated B cells; NLRP3, NOD-, LRR- and pyrin domain-containing protein 3; PCD, programmed cell death; RIPK, receptor-interacting protein kinase; RIPK1, receptor-interacting protein kinase-1; RIPK3, receptor-interacting protein kinase-3; ROS, reactive oxygen species.

**Figure 8A** showed the high-frequency keywords (n≥50) as an overlay map, where the color indicated the average published year. As we can see, inflammation, oxidative stress, phosphorylation, and protection are emerging fields that were colored yellow. The timeline view (**Figure 8B**) presented the top 3 (if any) high-frequency keywords in each cluster over time. We could see that seven of the eight clusters (except #6) are still ongoing. Among them, #0 (reperfusion injury/rat model) is the biggest cluster, followed by #1 (cell death/anticancer effect), #2 (necroptotic cell death/molecular switch), and #3 (oxidative stress/smoke-induced necroptosis). More information was listed in **Supplementary Table 2**.

**Figure 9:**
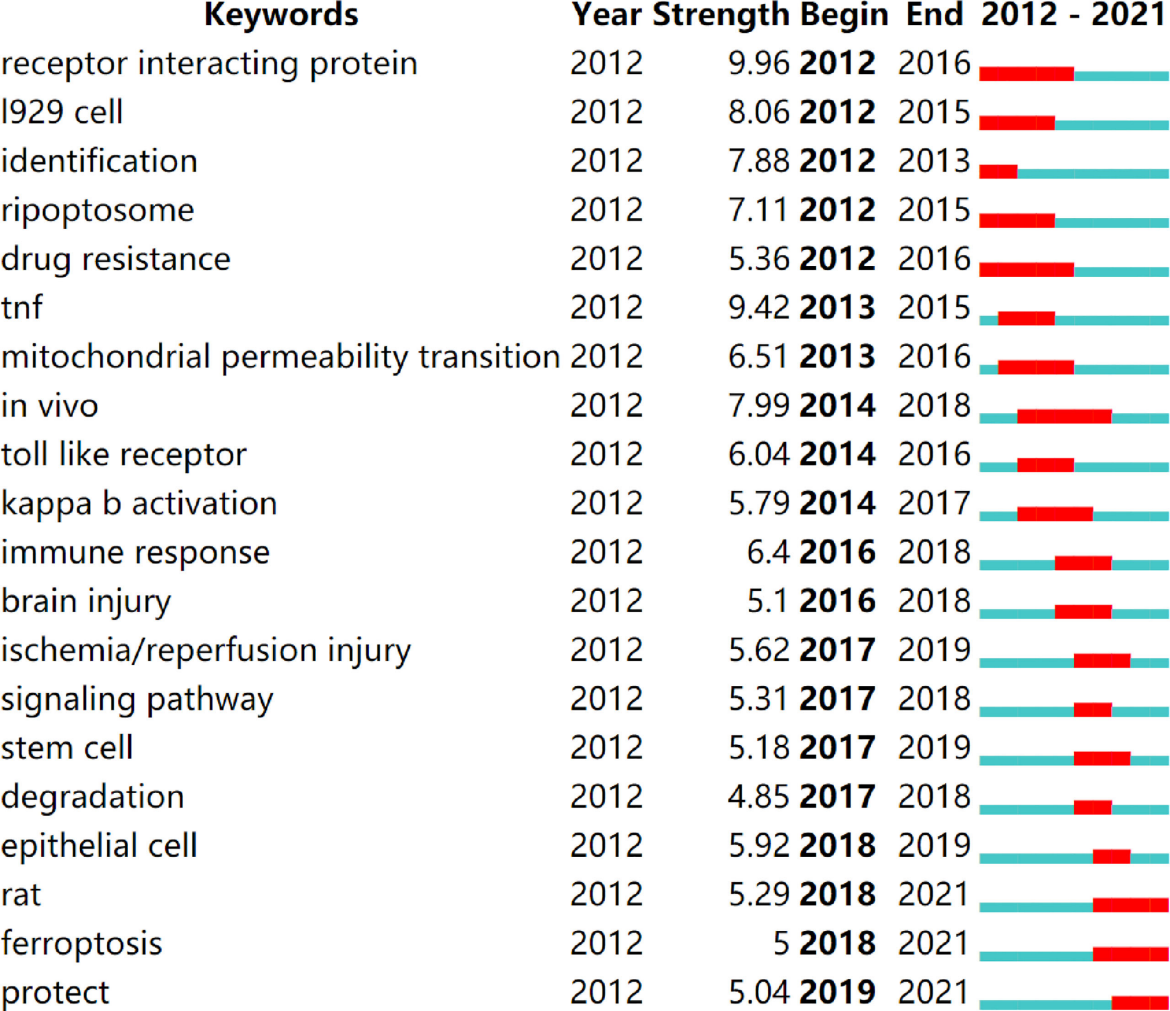
Top 20 keywords with the strongest citation bursts (sorted by the starting year). The red bars mean citation burstness.

Keyword bursts are those that were cited significantly frequently over a period (28). As shown in **Figure 9**, receptor interacting protein had the strongest bursts (strength=9.96), followed by TNF (strength=9.42) and l929 cell (strength=8.06). Notably, rat, ferroptosis, and protect were in burstness until 2021.

## 4 Discussion

### 4.1 General Information

Based on the data from the WoSCC database from 2012 to 2021, a total of 3,111 necroptosis articles were published in 786 academic journals by 19,687 authors in 885 institutions from 82 countries/regions.

The increasing trend of publications indicated that necroptosis is attracting mounting attention and interest. The necroptosis research officially started in 2005, when Junying Yuan proposed the term “necroptosis” (3). Since then, necroptosis research has grown rapidly. In the past decade, necroptosis research had stable stepwise growth, and the published article in 2021 is almost ten times that of 2012.

In country/region analysis, the number of publications and betweenness centrality are two vital indicators, in which high centrality (≥0.10) nodes imply the “bridge” effects of those countries/regions in the global cooperation network (26, 27, 42, 43). According to **Table 1** and **Figure 3**, China and the United States contributed the most to publications on necroptosis. Of the top 10 institutions that published the most research items, five were in China, two in the United States, two in Australia, and one in Belgium (1/10). However, the centrality of China and Chinese institutions was less than 0.1 while that of the United States was 0.23, indicating that the United States might maintain the dominant position in necroptosis research. Moreover, Germany, the United States, and France had high centrality, which means they played key roles in the global cooperation of necroptosis research. Furthermore, in terms of network density, there was active cooperation among countries and institutions.

Among the top 10 authors and co-cited authors (**Table 2**), Peter Vandenabeele not only published the greatest number of necroptosis-related papers but is also among the top seven co-cited authors, indicating his outstanding contribution to necroptosis research. Vandenabeele is a professor at Ghent University, focusing on cell death, immunology, and inflammation. In 2010, his group published a review (36) that described the molecular mechanisms of necroptosis in detail and discussed its immunological outcomes and pathophysiological implications, which was co-cited up to 542 times and had the strongest citation bursts in this study. Among both top 10 authors and co-cited authors, Andreas Linkermann is also a professor working in Technische Universitat Dresden; his laboratory is interested in multiple regulated cell deaths in kidney transplantation, acute kidney injury, antibody-mediated rejection, and ischemia-reperfusion injury. In 2014, the *New England Journal of Medicine* published a Linkermann and Green’s review entitled Necroptosis (1). They summed the regulatory mechanism and pathophysiology of necroptosis and pointed out the therapeutic strategies for preventing necroptotic diseases; this reference had a strong burst from 2015 to 2019. Notably, the most co-cited author, Alexei Degterev, an associate professor at Tufts University, recently centered on analyzing the roles of RIPK1/RIPK3 in necroptosis and inflammation. He published two top 10 co-cited articles that proposed the concept “necroptosis” (3) and identified the RIP1 kinase as a specific cellular target of necrostatins distinct from necrostatin-1 (38), respectively.

In a journal analysis (**Table 3**), *Cell Death & Disease* published the most necroptosis studies, and it was also the eighth most cited journal. *Cell death and differentiation* and *PNAS* were both the top 5 publication journals and top 5 co-cited journals, indicating their essential role in disseminating necroptosis research. *Cell* received the most co-citations, partly because three of the top four highly cited papers (33–35) were published in *Cell* (**Table 4**), followed by *Nature* and *PNAS*. The journals were mainly in the cell biology, immunology, and comprehensive fields. This is consistent with the dual-map analysis (**Figure 5**), which showed that the main citation path in necroptosis research is related to molecular, biology, and immunology.

The collection of co-cited references cited by the corresponding research community could partly represent the knowledge base (28, 44–46). Among the top 10 co-cited references, six mainly elaborated RIPK1 or RIPK3 (33–35, 37–39), three are about the key role of MLKL (33, 39, 40), two are related to the necroptosis in inflammation (17, 34), one prompted the term “necroptosis” and regarded it as a therapeutic potential for ischemic brain injury (3), and one reviewed the molecular mechanisms of necroptosis (36). As for reference burst analysis, four references are still in burst and worth our attention: three are about necroptosis-related diseases and inflammation (16–18), and one is a recommendation on the molecular mechanisms of cell death (41).

### 4.2 The Hotspots and Trending

In such an era of information explosion, keeping abreast of the trending research area in the research field is critical to researchers. Bibliometrics provides a method in which keyword co-occurrence can reflect the hotspots of an academic area (47), the overlay and timeline view can present the evolution of new hotspots (21, 48), and reference clusters and citation bursts can characterize the emerging topics in the discipline (23, 28, 42). In this study, we tried to objectively evaluate the hotspots and frontiers of necroptosis research through the analysis of keyword co-occurrence (**Table 5** and **Table 6**), keyword overlay and timeline (**Figure 8**), keyword burst (**Figure 9**), reference timeline (**Figure 6**), and reference burst (**Figure 7**). We summarized three aspects as follows.

#### 4.2.1 The Cross-Talk of Necroptosis With Other Types of Cell Death

PCD is a form of cell death that results from the activation of signal transduction modules, including apoptosis, necroptosis, pyroptosis, and ferroptosis, and hence can be pharmacologically or genetically modulated (41). Over the last two decades, numerous studies have highlighted the cross-talk between multiple types of PCD rather than linear pathways with defined immunological outputs (49, 50).

The pathways of necroptosis and apoptosis are tightly linked through caspase-8 (51, 52), which not only is a typical activator of extrinsic apoptosis but also inhibits necroptosis through the cleavage of RIPK1 and possibly RIPK3 (53, 54). That is, the cell commits three fates when TNF engages its receptor and forms Complex I, which consists of TNFR1-associated death domain protein (TRADD), TNFR-associated factors (TRAF), RIPK1, the cellular inhibitor of apoptosis protein 1 (cIAP1), and the cellular inhibitor of apoptosis protein 2 (cIAP2) (55). If RIPK1 is ubiquitinated, cell death is aborted; if caspase-8 activity is inhibited, necroptosis occurs; otherwise, the cells undergo apoptosis (56).

Necroptosis and pyroptosis are both regulated necrosis, among which pyroptosis can be initiated by the NLRP3 inflammasome when it suffers changes in cellular ion homeostasis, while this property also allows its activation in response to membrane disruption caused by MLKL, the terminal effector of necroptosis (57, 58).

Ferroptosis is an iron-dependent PCD caused by unrestricted lipid peroxidation and subsequent membrane damage (59). In this study, the keyword ferroptosis is not only in keyword co-occurrence; especially, it is also among the top 20 keywords with the strongest citation bursts and still on bursts and is the cluster label (#4) of the reference timeline (**Figure 6**) in which the cluster remains ongoing. That means ferroptosis still occurs frequently in recent necroptosis studies, indicating that the cross-talk between necroptosis and ferroptosis might be a new rising research area. Multiple structural, functional, and mechanistic evidence proved their cross-talk (60). One of the mechanisms of ferroptosis is that iron overload leads to a mitochondrial permeability transition pore (MPTP) opening, which exacerbates RIPK1 phosphorylation and contributes to necroptosis (61, 62). Moreover, heat shock protein 90 (HSP90) facilitates necroptosis and ferroptosis by promoting RIPK1 phosphorylation and inhibiting glutathione peroxidase 4 (GPX4) activation (63, 64). The cross-talk between necroptosis and ferroptosis has drawn attention in the areas of ischemic stroke (60), neuronal death after hemorrhagic stroke (65), kidney disease (66), pulmonary disease (67), and so on, which might have huge prospects.

Autophagy is also frequently mentioned in necroptosis research. Necroptosis and autophagy could cross-talk through RIPK1. When RIPK1 activates ERK, ERK negatively regulates transcription factor EB (TFEB) and, subsequently, basal autophagy through phosphorylation at the serine 142 site; if RIPK1 forms DISC and caspase-8 is activated, it leads to necroptosis (68).

Recently, a review discussed the emerging connectivity of PCD pathways and its physiological implications (50). The authors pointed out that the various types of PCD might be a single, coordinated cell death system in which the individual pathways are highly interconnected and can flexibly compensate for one another. Similar to our findings, the cross-talk between necroptosis and other cell death modalities and the synergy in disease might be the trending research area in the field of necroptosis.

#### 4.2.2 Potential Applications of Necroptosis in Various Diseases

According to our results (**Table 6**, **Figures 6**–**9**), necroptosis-related disease is a hot area in necroptosis research, including cancers (cancer, tumor, carcinoma, breast cancer, hepatocellular carcinoma, leukemia, drug resistance), neurological diseases (AD, brain injury, stroke, PD, ischemia/reperfusion injury), acute kidney injury, other ischemia/reperfusion injuries, infection, autoinflammatory disease, and myocardial ischemia. We discussed the first two fields below: cancers and neurological diseases.

The role of necroptosis in cancer is still controversial (6, 69). On the one hand, the dysregulation of necroptosis regulatory molecules such as MLKL and RIPK3 is associated with cancer development (70–72), and the activation of RIPK1/RIPK3 may potentiate antitumor immunity (73) and reverses drug resistance (70). On the other hand, there is also evidence that necroptosis may promote carcinogenesis by inducing adaptive immunosuppression; for instance, RIPK1 is overexpressed in glioblastoma, lung cancer, and pancreatic ductal adenocarcinoma (PDAC) (74, 75), and RIPK3 and MLKL are highly expressed in PDAC (75, 76). Among the clinically approved drugs, 5-FU (77, 78) and shikonin (79, 80) might enhance antitumor immunity through necroptosis induction. Overall, although the molecular mechanism of necroptosis has been studied well, its application and regulation in cancer only began to emerge and need further investigation (20).

Similar to our findings, accumulating evidence indicates that age-related neurodegenerative diseases, such as AD and PD, and acute neuronal injury, such as stroke, traumatic brain injury, and ischemia/reperfusion injury of brain, are strongly associated with necroptosis (8, 81, 82). Notably, degradation is among the top 20 keywords with the strongest citation bursts (**Figure 9**), indicating that it is a hot research field from 2017 to 2018; moreover, neuronal cell death is extracted as the label of cluster #5 (**Figure 8B**) that is still ongoing, meaning it remains as the research foci for now. AD is characterized by severe neuronal loss in which necroptosis is observed and correlated positively with the Braak stage and negatively with brain mass and cognition (8, 83). RIPK1, a key regulator of necroptosis, has emerged as a promising therapeutic target for neurodegenerative disease (81, 84, 85). It might be involved in regulating transcriptional responses in AD, and the inhibition of RIPK1 might promote the ability of microglia to degrade amyloid-β, reduce inflammatory microglia, and restore the phagocytic capacity of microglia (84). Recently, Park et al. demonstrated that O-GlcNAcylation (O-linked β-N-acetylglucosaminylation) plays a protective role in AD by inhibiting necroptosis through ameliorated AD pathology, including Aβ burden, neuronal loss, neuroinflammation, and damaged mitochondria and recovered the M2 phenotype and phagocytic activity of microglia (86). The increased levels of RIPK1, RIPK3, and MLKL were also observed in the PD model (81); furthermore, the necrostatin-1, an inhibitor of necroptosis, improves the survival of optic atrophy type 1 mutant human iPSC–derived neurons *in vitro* and attenuate MPTP-induced dopaminergic neuron loss (9). Necroptosis was also observed in traumatic brain injury, stroke, and ischemia/reperfusion injury of brain (87–89). Moreover, the inhibitor of necroptosis, necrostatin-1, has been regarded as a promising treatment target for neurodegenerative diseases (88, 90, 91). Necroptosis has shown a great therapeutic promise in neurodegenerative diseases and acute neuronal injury, which has attracted the researchers’ interests and is becoming a trending topic.

#### 4.2.3 Mechanism of Necroptosis

As shown in **Tables 5**, **6**, in the necroptosis research field, the hotspots of key players include RIPK1, RIPK3, and MLKL; the hotspots of triggering factors include TNF-alpha, NF-κB, toll-like receptors, and ZBP1, as supported by the timeline analysis (**Figures 6**, **8**). We will not repeat them because previous reviews (1, 36, 92) have thoroughly explained these. Notably, our results show that inflammation and oxidative stress are at the forefront of the current necroptosis research.

It is known that necroptosis is an inflammatory form of PCD; when cells die and the membrane ruptures, damage-associated molecular patterns (DAMPs) that can cause inflammatory responses are released (18, 93). In addition, previous studies suggested that RIPKs facilitate the activation of the NLRP3 inflammasome (94, 95). Indeed, necroptosis is associated with some inflammatory diseases, such as neuroinflammatory disease (96), infection (12), autoinflammation (97), inflammatory bowel disease (13), and idiopathic inflammatory myopathy (98). Therapeutically, the inhibition of RIPK3 or RIPK1 exhibited anti-inflammatory effects in animal disease models, suggesting that the inhibitors of these kinases may have a therapeutic potential to treat inflammatory injuries (99).

Oxidative stress is labeled as the fourth cluster that is still ongoing (**Figure 8**, #3); it is an imbalance between oxidants and antioxidants in favor of the oxidants, leading to a disruption of redox signaling and control and/or molecular damage (100). Oxidative stress is caused by an imbalance between the production of ROS and the antioxidant capacity. Oxidative damage is not only the cause of necroptosis but also its consequence (101, 102). Excess ROS leads to lipid peroxidation and damage to proteins and DNA, and the latter is an important cause of genomic instability in age-related diseases (102). For instance, oxidative stress could promote Aβ deposition, tau hyperphosphorylation, and synaptic and neuronal loss and subsequently contributes to the development of AD (103).

### 4.3 Strength and Limitations

Overall, this is the first bibliometric study to systematically analyze the necroptosis-related publications in the past decade. Compared to traditional reviews, the bibliometric analysis provides a novel and objective insight into the evolving research foci and trends (24). Meanwhile, we used various bibliometric software to perform an analysis, which could provide richer results in multiple dimensions (24, 104). This study will inform the public of the importance of necroptosis, provide scholars a whole picture of necroptosis research, and further serve as a comprehensive and objective guide for the future development of the necroptosis research field.

Inevitably, this study has some limitations. Firstly, we exclusively retrieved the articles published in English from the WoSCC database, thus omitting articles that are not in WoSCC or not English. Nevertheless, English articles in WoSCC are the most commonly used data source in bibliometrics, which could represent most of the information to a degree (24, 105). Secondly, bibliometric methods are based on natural language processing, which may be biased, as reported by other bibliometric studies (21, 24). However, our results are consistent with recent traditional reviews (92, 106, 107) while providing more comprehensive and objective information.

## Conclusion

In conclusion, research on necroptosis had a stable stepwise growth with active cooperation worldwide in the past decade, of which the United States might maintain the dominant position in necroptosis research. Peter Vandenabeele contributed to most of the publications, and Alexei Degterev was the most co-cited in necroptosis field. Current necroptosis studies are focused on its cross-talk with other types of cell death, potential applications in disease, and further mechanisms. Among them, the synergy with ferroptosis, further RIPK1/RIPK3/MLKL studies, the mechanism and translational applications with inflammation and oxidative stress, and the therapeutic potential to treat cancer and neurodegenerative diseases might be the rising and promising research areas. These might provide guidance and new insight for further research in the necroptosis field.

## Data Availability Statement

The original contributions presented in the study are included in the article/**Supplementary Material**. Further inquiries can be directed to the corresponding authors.

## Author Contributions

HX, JQJ, JL, and JZ designed this study. JDJ and WT collected and cleaned the data. JZ and LS performed the analysis. RL normalized the pictures. ZZ and JDJ re-checked data. JZ and LS wrote the original draft. All authors reviewed the manuscript.

## Funding

The work was supported by the National Natural Science Foundation of China (No. 82004145, 82004301, 81874412), CACMS Innovation Fund (CI2021A00917), Central Public Welfare Research Institutes of China Academy of Chinese Medical Sciences (No. ZZ13-YQ-017), and the Fundamental Research Funds for the Central Universities, Beijing University of Chinese Medicine (No.2022-JYB-XJSJJ-053).

## Conflict of Interest

All authors declare that the research was conducted in the absence of any commercial or financial relationships that could be construed as a potential conflict of interest.

## Publisher’s Note

All claims expressed in this article are solely those of the authors and do not necessarily represent those of their affiliated organizations, or those of the publisher, the editors and the reviewers. Any product that may be evaluated in this article, or claim that may be made by its manufacturer, is not guaranteed or endorsed by the publisher.

## Supplementary Material

Supplementary Table 1 Included studies (.xlsx).

Supplementary Table 2 Additional information of figures (.xlsx).

Supplementary Table 3 High-frequency co-cited references (.xlsx).
